# Enhanced annotations and features for comparing thousands of *Pseudomonas* genomes in the Pseudomonas genome database

**DOI:** 10.1093/nar/gkv1227

**Published:** 2015-11-17

**Authors:** Geoffrey L. Winsor, Emma J. Griffiths, Raymond Lo, Bhavjinder K. Dhillon, Julie A. Shay, Fiona S. L. Brinkman

**Affiliations:** Department of Molecular Biology and Biochemistry, Simon Fraser University, Greater Vancouver, BC V5A 1S6, Canada

## Abstract

The Pseudomonas Genome Database (http://www.pseudomonas.com) is well known for the application of community-based annotation approaches for producing a high-quality *Pseudomonas aeruginosa* PAO1 genome annotation, and facilitating whole-genome comparative analyses with other *Pseudomonas* strains. To aid analysis of potentially thousands of complete and draft genome assemblies, this database and analysis platform was upgraded to integrate curated genome annotations and isolate metadata with enhanced tools for larger scale comparative analysis and visualization. Manually curated gene annotations are supplemented with improved computational analyses that help identify putative drug targets and vaccine candidates or assist with evolutionary studies by identifying orthologs, pathogen-associated genes and genomic islands. The database schema has been updated to integrate isolate metadata that will facilitate more powerful analysis of genomes across datasets in the future. We continue to place an emphasis on providing high-quality updates to gene annotations through regular review of the scientific literature and using community-based approaches including a major new *Pseudomonas* community initiative for the assignment of high-quality gene ontology terms to genes. As we further expand from thousands of genomes, we plan to provide enhancements that will aid data visualization and analysis arising from whole-genome comparative studies including more pan-genome and population-based approaches.

## INTRODUCTION

*Pseudomonas* is a metabolically diverse genus of bacteria known for its members’ ability to thrive in multiple environments including soil and water, contain intrinsic antimicrobial resistance and/or act as opportunistic pathogens of humans, animals and plants. Owing to a large, conserved core region and a very diverse catalog of accessory regions, and their impressive metabolic capacity, the study of *Pseudomonas spp*. genomes and their role in shaping these lifestyles is an intense focus of interest (1,2). With recent advances in whole-genome sequencing, thousands of complete and draft *Pseudomonas* genome assemblies are being rapidly generated from environmental and clinical isolates, making it critical to store data in a format suitable for facilitating downstream comparative analyses and distribution to the wider research community. Since 2000, the Pseudomonas Genome Database team and participating community members have contributed more than 2500 updates to *Pseudomonas aeruginosa* PAO1 genes and the website has facilitated comparative analyses for other completely sequenced *Pseudomonas* genome (3). This article highlights recent developments for the Pseudomonas Genome Database including significant updates to *P. aeruginosa* PAO1 gene annotations, an upgraded database schema, and other enhancements that facilitate better whole genome comparative analyses between the type strain PAO1 and other *Pseudomonas spp*.

## FOCUS ON COMMUNITY-ASSISTED, CONSERVATIVE GENOME ANNOTATION

With the sequencing of the *P. aeruginosa* PAO1 genome in 2000 (4), an intensive genome annotation effort was conducted by the *Pseudomonas* Community Annotation Project (PseudoCAP). Consisting of three curators and additional 61 researchers from 13 countries, PseudoCAP was the first successful community-based genome annotation effort conducted entirely via the internet (5). It resulted in 1741 updates using a model where in-house literature review was complemented by community-based submission of annotations via web forms or email that were subject to a secondary review step to ensure consistency. Since 2000, a similar approach involving two part-time curators and 106 researchers from 20 countries has been used to add an additional 2900 annotations to the database. While more than 2200 *P. aeruginosa* PAO1 genes have been critically and conservatively annotated to date, this is an ongoing process and it is important to note that a similar number of genes remain annotated as ‘hypothetical proteins.’

### *Pseudomonas* GO term annotation initiative

A recent focus for the database team has been on mapping *P. aeruginosa* PAO1 gene annotations to terms from the Gene Ontology (GO); a hierarchical controlled vocabulary representing three major domains of biological information: (i) cellular component, (ii) molecular function and (iii) biological process (6). There are several advantages to having genes mapped to GO terms including less ambiguity, easier sharing of data and the ability to perform more sophisticated analysis of experimental data (e.g. RNA-Seq data) using such methods as gene set enrichment analysis to identify relevant biological domains associated with a given phenotype. While all completely sequenced genomes in the database are assigned GO terms based on mappings to InterPro functional domain predictions (7), we recognize the importance of annotating genes with high-quality GO terms using the rich information available from *P. aeruginosa* experimental studies. Since performing such a task is a time-consuming process, we coordinated a community-based GO term annotation initiative in 2014 where members of the *P. aeruginosa* research community and an in-house team curated 3533 GO term annotations for *P. aeruginosa* PAO1.

### Evidence ontology

The degree of granularity for evidence describing the experiments that GO terms are based on was also enhanced by assigning Evidence Ontology (ECO) codes (8). Therefore, users can utilize a complete set of *P. aeruginosa* PAO1 GO terms (or a filtered set of higher quality terms based on ECO codes) for use with third-party analysis applications by downloading them in GO Annotation File (GAF) Format 2.0.

### Curated virulence factor annotations

Another focus of the database has been on integrating high-quality data on virulence factors from sources including the Virulence Factor Database (9), Victors Virulence Factors from the PHIDIAS database (10) and our own curation. We recognize that virulence is very context-dependent, so we have made an additional effort to assign more contextual information including ECO codes, host organism and infection models to our own annotations as well as externally curated annotations.

## WHOLE GENOME COMPUTATIONAL ANALYSES THAT COMPLEMENT CONTINUALLY UPDATED ANNOTATIONS

We also complement our continually updated, curated annotations with a range of computational methods to help identify suitable targets for vaccine candidates or drug targets or otherwise aid characterization of protein features and identify genes that contribute to a pathogenic lifestyle. The results of these analyses are available from any gene details page or from a variety of other specialized listings of the analyses, and include the following that have been expanded:

### PSORTb protein subcellular localization prediction/identification of cell surface/secreted proteins

For deduced proteins from all *Pseudomonas* genomes, PSORTb 3.0 prediction of protein subcellular localization now contains new localization subcategories suited for identifying proteins associated with the Type III secretion apparatus, fimbria, flagella or proteins that are targeted to a host cell (11). Subcellular localization data can be accessed via a proteome-wide breakdown of predicted and experimentally validated localizations made available from individual strain overview pages, strain-specific lists of proteins and their subcellular localization annotations, or they can be added as a condition into any advanced search. Such protein localization information is valuable for prediction of protein function and identification of cell surface or secreted proteins of interest as potential diagnostic targets, vaccine components or drug targets.

### Proteins with 3D structures

We identify proteins that have greater than 90% similarity to proteins found in the RCSB Protein Data Bank (12). A specialized page containing a list of all proteins identified using this method can be accessed under the ‘Annotations By Category’ section of the top menu. Also, having such similarity to a protein with a known 3D structure can be applied to any advanced search.

### Functional predictions using InterProScan 5

To identify potential functional domains in proteins from completely sequenced genomes, we utilize the InterProScan 5 software package that takes as input protein sequences to search against InterPro's various signatures provided by different databases including PROSITE, PFAM, PRINTS, SMART, TIGRFAMs, etc. (7). The results of this analysis can be accessed as a list of functional domain annotations assigned to each of the protein-coding genes for a given *Pseudomonas spp*. strain, through a tab on the details page of a protein-coding gene or visualized in the context of the order they occur in a protein relative to the genome sequence by using JBrowse.

A benefit of the InterPro approach is that once hits are found and associated with an InterPro entry, InterProScan 5 links them with KEGG pathways (13), UniParc pathways (14) and GO terms which can be used for genome wide analysis in strains where a curated list of data is not available. In the case of reference strains such as PAO1 or PA14, evidence codes are used to distinguish the InterPro-mapped pathways and GO terms from the manually curated pathways and terms; this allows you to remove InterPro-mapped pathways/GO terms from your list if necessary.

### Ortholog predictions and *Pseudomonas* ortholog groups (POGs)

Further computational analyses of protein sequences include our high-quality ortholog and orthologous group predictions using the Ortholuge approach for evaluation of putative orthologs. It is based on reciprocal best BLAST analysis being subjected to a further phylogenetic analysis step where the phylogenetic distance ratios of two in-group proteins (the reciprocal best BLAST hits) relative to an outgroup protein are compared to the phylogenetic distance ratios of the same isolates as a whole (15,16). As an alternative, orthologous group classifications such as Clusters of Orthologous Groups of proteins, COGs (17) that some *Pseudomonas* proteins cannot be classified into, we provide a separate analysis that assigns *Pseudomonas* proteins to more inclusive groups of orthologs and putative paralogs called *Pseudomonas* Ortholog Groups based on a method previously described (18).

### Pathogen-associated genes

A relatively new analysis added to the site (and last updated in May 2015) is our pathogen-associated genes analysis based on a previous analysis approach developed in our lab (19) whereby BLAST similarity searches were performed against the deduced proteomes of completely sequenced bacterial genomes obtained from the National Center for Biotechnology Information (NCBI) FTP site (20) and incorporated into our MicrobeDB database (21), with knowledge of the pathogenicity of each bacterial isolate manually curated as being either nonpathogenic or pathogenic (i.e. pathogen of any organism, from human, to other animal to amoeba). The output of the analysis for each protein is a classification indicating whether it is pathogen-associated (i.e. the protein disproportionately had similarity at a certain cut-off to proteins only in pathogens), nonpathogen-associated (protein only had similarity at the cut-off to proteins in nonpathogens) or common (protein had similarity to proteins in both pathogens and nonpathogens). It is important to note that genes identified as being pathogen-associated will change as more genomes are being sequenced and so this analysis must be regularly updated.

### Genomic islands, with overlaid virulence and antimicrobial resistance gene information

An area of intense focus in *Pseudomonas* genomics is based on gaining a better understanding of core versus accessory genome content, and how the horizontal transfer of mobile genetic elements (including phage and transposons) contributes to the evolution of antimicrobial resistance, virulence and adaptation to growth in new environments. Collectively, any cluster of genes of potential horizontal origin (including phage, usually at least 8 kb in length;(22)) are known as genomic islands (GIs). GIs display many characteristics that can distinguish them from the core genome, including differences in GC content, dinucleotide bias and the presence of mobility genes (e.g. transposases and integrases) or higher percentages of hypothetical protein genes (22,23). We therefore integrated the new IslandViewer 3, released in 2015, which is comprised of GI predictions detected using the three most precise computational methods that identify GIs using different, but complementary approaches (24). GI predictions can be viewed from links to the IslandViewer website on overview pages for individual chromosomes/plasmids or by going to JBrowse and selecting the ‘Genomic Island Predictions’ track from the left panel. Note that pathogen-associated genes can also be viewed in JBrowse alongside the predicted GIs and the link from JBrowse to the IslandViewer website shows these genes in the context of genomic islands, curated virulence factors and antimicrobial resistance genes identified from the Antibiotic Resistance Genes Database (25) and Resistance Gene Identifier predictor (26).

### Other miscellaneous predicted sequence features

As a complement to manually curated transcriptional units, we provide computationally predicted rho-independent transcription terminators based on TransTermHP (27). We also provide inverted repeats identified using the palindrome program from EMBOSS (28) for all isolates in the database which can be useful for identification of regulatory regions such as riboswitches (29) and sites responsible for mediating genome rearrangements (30).

## UPDATED WEBSITE AND DATABASE FUNCTIONALITY WITH ENHANCED VIEWS

**Figure 1.**
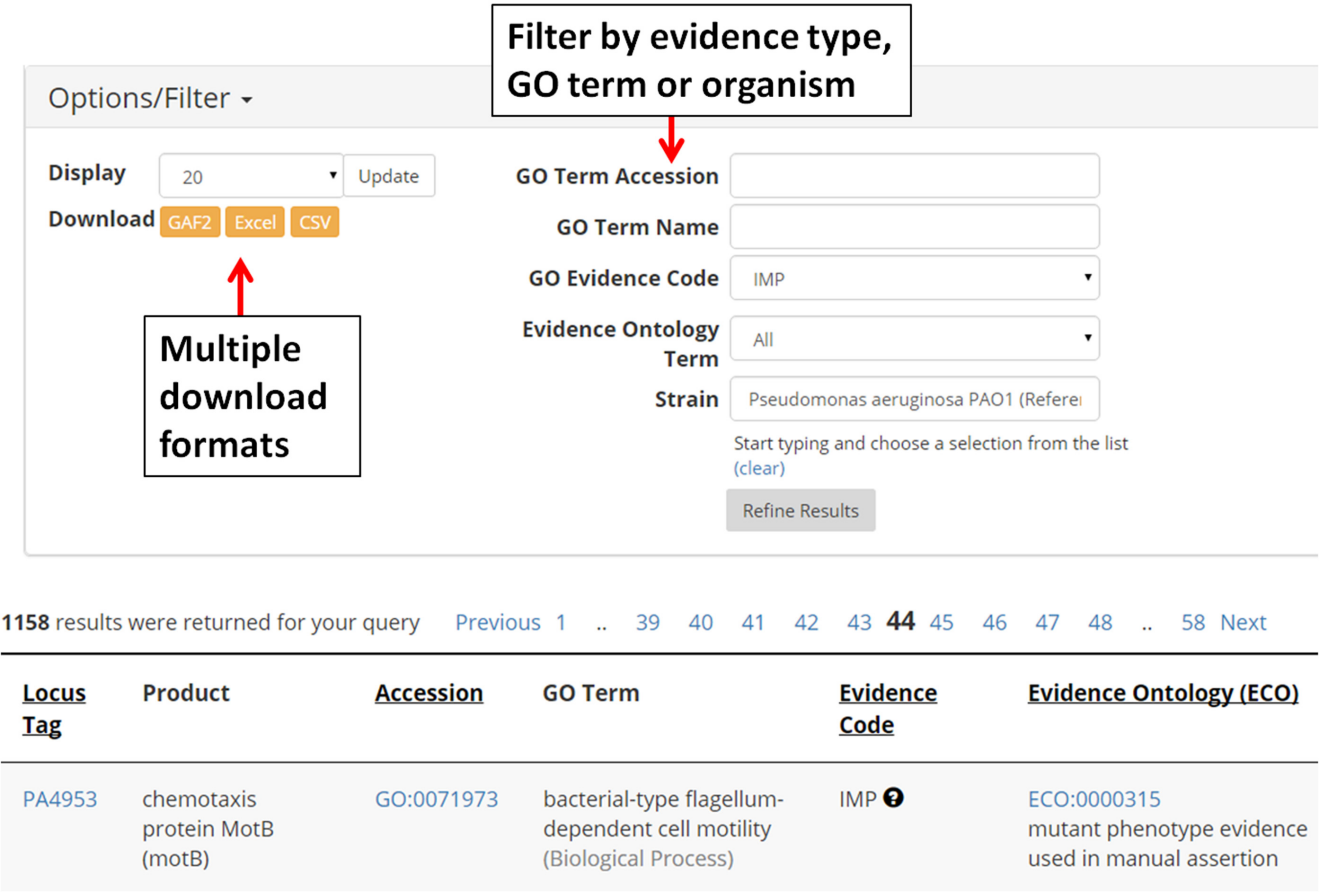
A representative view of the enhanced display of genome-wide data tailored to specific annotation types. In this example, Gene Ontology (GO) term annotations for *Pseudomonas aeruginosa* PAO1 can be customized to provide distinct subsets of data based on specific GO terms or types of evidence (e.g. based on GO or ECO evidence codes). Results can be downloaded in GO Annotation File 2.0 format suitable for downstream applications including differential expression analysis.

We have recently released an updated beta version of the Pseudomonas Genome Database website that maintains all existing functionality from our previous version (3) and provides improvements that make relevant whole-genome datasets more readily available, streamlines the way users navigate them and provides a range of useful download options suitable for downstream applications. For example, we have created a drop-down menu on the front page providing links to a range of datasets featuring genome-wide analyses and other functional annotations including the computational analyses mentioned above (e.g. virulence factors, GO annotations, pathways, COGs, etc.). The results returned for each analysis can easily be customized to provide distinct subsets of data based on specific strains, functional terms, source database and types of evidence including GO evidence terms and ECO evidence codes (Figure 1). For the results of a simple or advanced keyword search, one can also customize the displayed columns in order to add various fields including protein features such as molecular weight and isoelectric point or various external cross-references including RefSeq accession, UniProtKB accession, GI or mapped relationships between old versus new locus tags assigned by NCBI. Once a list of results has been obtained by an advanced search, a user can perform various subqueries of the data using criteria specific to the search. For example, when viewing a list of GO terms assigned to genes in *P. aeruginosa* PAO1, one can filter the results to only include manually curated annotations based on mutant phenotype evidence using either GO consortium evidence codes or evidence ontology terms. In addition, results pages also provide new options for downloading data in formats suitable for downstream analysis outside of the Pseudomonas Genome Database platform. For example, GO term annotations can be downloaded in GAF 2.0 format compatible with tools used for gene set enrichment analysis, while gene lists generated from an advanced search of genome annotations can be downloaded in formats including CSV, TAB, GTF and GFF3 for use with other external applications.

Via the new IslandViewer 3, enhanced visualizations of genomes are also provided using a newly developed genome viewer based on GenomeD3Plot software (31). This visualization provides an interactive, flexible user experience, using the JavaScript D3 library, enabling viewing of regions of genomes in a dynamically displayed circular genome viewer with corresponding vertical and horizontal genome views to enable easier visualization of gene annotations and gene arrangements/context. This viewer integrates GI, antimicrobial resistance, virulence factor, pathogen-associated genes analysis and protein/gene annotations, also allowing comparisons between two genomes.

Individual gene detail pages have been updated with additional annotation details and links to other views and analyses. For example, the *P. aeruginosa* PAO1 oprF annotation gene overview now contains new experimental evidence for its translation and subcellular localization based on multiple studies, a new link to predicted protein–protein interactions in the STRING database (32), new database cross-references not found in the older version of the database and links to a new genome browser (JBrowse) that complements our older GBrowse view and contains more dynamic visualization capabilities. The links to known PAO1 transposon mutants have been updated to also include known transposon insertions in *P. aeruginosa* PA14 orthologs. A ‘Functions/Roles’ tab on the details page will bring you to GO terms assigned by manual curation and sequence similarity, with rich details describing the type of evidence the annotations are based on, whether they were subject to manual review by a curator, and links to the primary literature. The same page will also provide PseudoCAP functional classifications and predicted functional domains based on InterProScan 5 described above. A new ‘Expression Data’ tab has also been added to the gene details pages and currently displays expression profiles obtained from the Gene Expression Omnibus (GEO) database at NCBI (20). GEO profiles allow one to view relative changes in gene expression across a range of conditions and link directly to analyses at NCBI showing genes with similar expression profiles from the same dataset. The ‘Motifs’ tab links to an updated page containing a list of all motifs that have been annotated in that gene, as well as any relevant motifs in the upstream or downstream intergenic regions. We integrate a wide range of sources for motifs including PRODORIC (33), RegTransBase (34), CollecTF (35), inverted repeats and rho-independent terminators, as well as our own in-house curation. Details on regulatory regions can also be accessed from clickable links on the gene map at the top of each gene details page which will bring the user to a customized intergenic region overview containing a list of curated and predicted motifs, nucleotide sequences for the forward and reverse strand of the intergenic region, and a form for updating the amount of sequence shown (in case you wish to expand the viewable area into the upstream or downstream ORFs that are surrounding the region). With respect to curated or predicted motifs, we have also included new functionality to look for specific or nonspecific variations of a motif occurring in the same or other genomes using an updated version of our DNA motif search tool that is built on top of the fuzznuc program developed by EMBOSS (28). This is a useful alternative to using BLAST or other heuristics to search for occurrences of small motifs in other genomes.

The site also provides other options for searching for matches to a specific sequence with the most widely used tool being BLAST (36). New changes to the search interface allow users to query BLAST nucleotide or protein sequence databases at the individual strain, species or genus level while also specifying whether draft genomes should be included in the search. The results of a BLASTP search will include links to the specific gene coding for the protein while BLASTN search results will contain links to a view of the hit in context of the local genome via JBrowse, GBrowse or a view similar to the Pseudomonas Genome Database gene and intergenic region details pages.

### New ways to visualize genome annotation, RNA-seq and variant data

**Figure 2.**
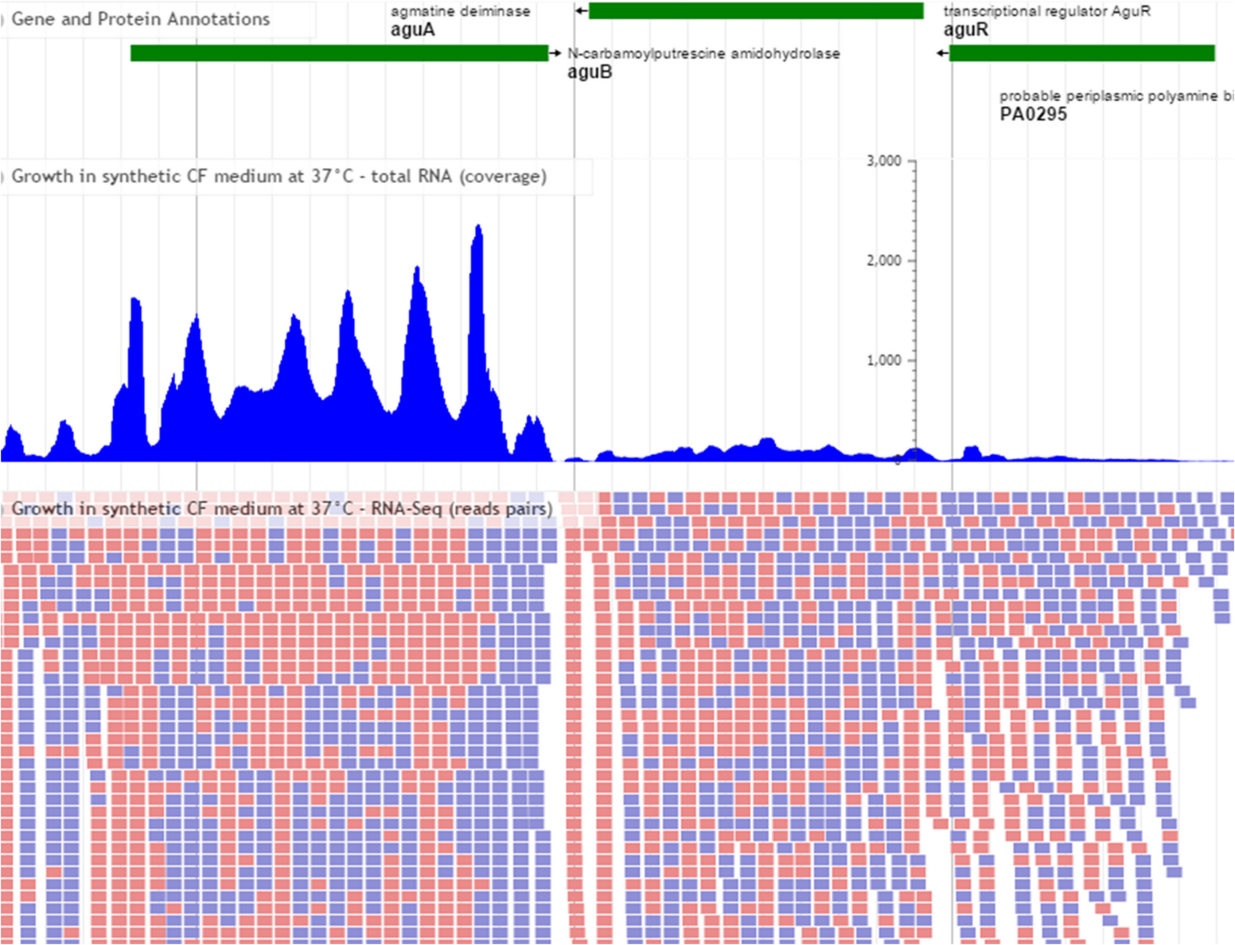
A wide range of genomic features are available for visualization through the Pseudomonas Genome Database implantation of GMOD's JBrowse viewer. This example shows an upper track containing glyphs corresponding to annotated *Pseudomonas aeruginosa* PAO1 genes, a middle XY-plot track representing RNA-Seq coverage based on an indexed bam file, and a lower track containing alignments of individual reads from the same file. Users can also open and view their own RNA-Seq data, variant files and GFF3 annotations in context of other up-to-date genomic features publicly available through the site. All data available through the interface can be further customized in the browser or downloaded in a range of different formats.

We provide multiple different views to graphically view annotations in the context of other sequence features in the genome. In addition to the new, interactive IslandViewer display, we continue to use GBrowse (37) as a viewer for all genomes available prior to the release of this latest version of this database. In addition we now use JBrowse (38) as the default viewer for all completely sequenced genomes. Using JBrowse, one can view a wide range of informative data tracks including continually updated gene and protein names, regulatory regions, functional domain predictions based on InterProScan 5 (7), genomic island predictions based on IslandViewer 3 (24), curated virulence factor genes and our pathogen-associated gene analysis while being able to link to more information at their associated websites. Users have the option to view and customize their own data tracks including feature annotations, XY coverage plots for RNA-Seq and stacked views of raw reads for variant data without having to upload large files to a remote server (Figure 2). Other views, such as our schematic view of genes at the top of any gene details card, continue to be available and PseudoCyc (39) metabolic pathways views continue to be maintained.

### Integration of more isolates and related metadata

There has recently been a marked increase, particularly with respect to draft genomes, in the number of *Pseudomonas* genomes being sequenced and appearing in the NCBI RefSeq database (40) and this trend will continue with the advent of large-scale genome sequencing projects including the *Pseudomonas* 1000+ Genome Sequence Project conducted by the International *P. aeruginosa* consortium (41). To date, most focus has been on sampling the physical environment and clinical settings to increase our understanding of genomic diversity. However, a growing proportion of studies utilize genome sequencing to better understand the evolution of diversity between spatial and temporal *P. aeruginosa* isolates such as in the cystic fibrosis airway (42). As of September 2015, the Pseudomonas Genome Database contains 1450 draft and 100 complete genomes with the number likely to continue increasing exponentially.

It is becoming apparent that sophisticated analysis of thousands of isolates derived from multiple datasets can only be facilitated by integrating more and better detailed metadata including details such as phenotype (e.g. serotype, antimicrobial resistance profile), geographical information including country, city and geographic information system coordinates as well as isolation source (e.g. sputum, burn, water, etc). We have updated the Pseudomonas Genome Database schema to store metadata for potentially thousands of complete and draft genomes based on the data models used by the BioProject, BioSample and Assembly databases at NCBI (20). Due to the sparse amount of information on some isolates, we also manually curate metadata using the Minimum Information about any (X) Sequence (MIxS) specification developed by the Genomic Standards Consortium (43) and used by the Genomes Online database (44) as a guide.

We encourage individual projects to take the initiative and structure their metadata with this in mind, however, as standards come into place, those accepting genome sequence submissions will need to adopt a leadership role and ensure they are adhered to. We also foresee the need for improvements to the tracking of metadata for sequential and temporal isolates or better linking of newly sequenced isolates to parental strains already in public repositories and have plans to address this matter in a future release. With respect to how metadata is utilized on the site, we have implemented initial functionality to allow filtering of strain listings criteria including antibiotic susceptibility/resistance, host organism and health status of the host (e.g. cystic fibrosis versus burn victim), and we anticipate it will be further exploited in the near future to facilitate better whole-genome comparative analysis (e.g. show genes found in cystic fibrosis isolates with resistance to a specific antibiotic versus all the other strains).

## CONCLUSION AND FUTURE NEEDS

We have described a collection of major improvements to the Pseudomonas Genome Database that facilitate improved comparison of thousands of complete and draft genomes, and more diverse, flexible visualizations of data, while continuing to place an emphasis on the importance of providing high quality genome annotations for representative strains/isolates. Note there is a ‘Latest News’ section on the website that effectively highlights all changes to the database since it was created. Moving forward, we see the need for more analyses that enable tree-based visualization of hundreds to thousands of isolates with phenotype or geographical information being overlaid. However, in order to conduct such sophisticated analyses across datasets, higher standards for metadata need to be adhered to by researchers and enforced by public repositories. In the near future, we will also need to focus on reducing the redundancy of annotations by clustering very similar genome sequences around highly curated representative genomes while more efficiently displaying sequence variants including single nucleotide variants and indels that will be most useful for outbreak and longitudinal studies. *Pseudomonas* researchers represent a very strong, active research community that are studying a collection of bacterial species of broad interest for their diverse metabolic capacity, ability to cause disease in plants and animals (or be beneficial) and in some cases contain intrinsic antimicrobial resistance to a broad range of antimicrobials that is of general interest. Development of a database that focuses on these medically, agriculturally and industrially important species, with customized data for this genus, complements other more general bacterial genome databases. It provides resource enabling analyses suitable at the genera level (e.g. robust ortholog comparisons, genera-specific annotations and analyses) that may be implemented for other genera (such as the Burkholderia Genome Database we also maintain). As there is growing appreciation that many genome-scale bioinformatics analyses need to be customized for a given genera or species, and that reference genomes for key phylogenetic clusters be maintained, such databases may become of increasing value in providing a portal to genomic analyses suitable for the genera, of use both to basic research and more applied clinical/outbreak analysis applications.
